# Hand-Foot-Mouth Disease in an Adult

**DOI:** 10.7759/cureus.33670

**Published:** 2023-01-11

**Authors:** Sara Gomes, Sara Santos, Inês Ferreira Maia, Rita Verissimo, Tiago Carvalho

**Affiliations:** 1 Internal Medicine, Centro Hospitalar Barreiro-Montijo, Barreiro, PRT; 2 Nephrology, Centro Hospitalar Lisboa Ocidental, Lisbon, PRT

**Keywords:** contagious infectious disease, hand-foot-mouth disease in adults, viral illness, enterovirus, hand-foot-mouth disease

## Abstract

Hand-foot-mouth syndrome is a common childhood illness. Although occurrence in adults is rare, its incidence has been increasing. In such cases, it usually presents with atypical symptoms.

The authors present the case of a 33-year-old male patient who presented with constitutional symptoms, feverish sensation, and macular palmoplantar rash associated with oral and oropharynx ulcers. The epidemiological history revealed exposure to two cohabitants (children) with a recent diagnosis of hand-foot-mouth disease (HFMD).

## Introduction

Hand-foot-mouth disease (HFMD) is a common, highly contagious infectious disease usually occurring during childhood (usually under 10 years old) [1,2]. It is most frequently caused by coxsackievirus-A16 and enterovirus-A71 with reports of coxsackievirus B1-B6 increasing in adults [2-6]. More recently, worldwide outbreaks of atypical presentation in adulthood associated with the coxsackie-A6 virus have been reported [4-6]. The atypical presentation may be characterized by high fever, a more varied distribution of lesions, marked skin involvement (with erosions, ulcers, and bullae), and a longer course of the disease, which may last two weeks [4,5].

Enteroviruses are responsible for a heterogeneous group of diseases and their transmission is usually by the fecal-oral route, ingestion of fecal material, and respiratory secretions containing the virus [4,2]. After ingestion, there is viral replication in the lymphoid tissue of the pharynx and intestine, spreading posteriorly [4]. According to some authors, most infections (90%) are subclinical, with authors describing clinical manifestations in less than 1% of adults [6]. The clinical presentation has a wide spectrum, from constitutional symptoms and rash to severe presentations that affect the central nervous system and the heart [4]. The most common clinical presentation is the vesicular rash on the palms and soles lasting seven to 10 days with subsequent desquamation [4].

## Case presentation

A 33-year-old male patient, a father of two children (aged three and one), presented with allergic rhinitis and Gilbert's syndrome. The patient had a personal history of chickenpox in childhood with no other relevant medical past.

**Figure 1:**
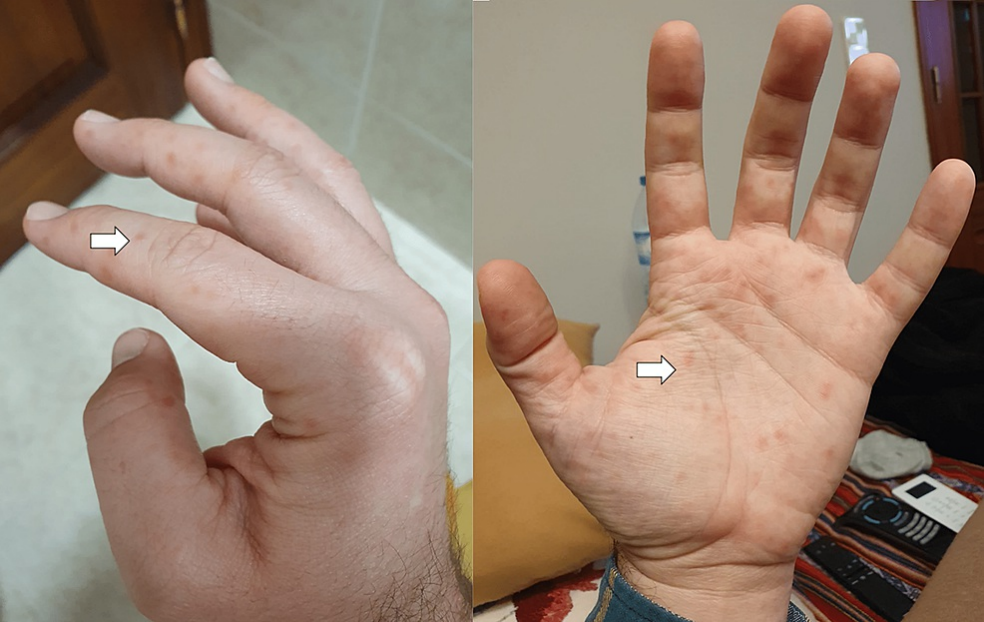
Macular lesions on the hands

**Figure 2:**
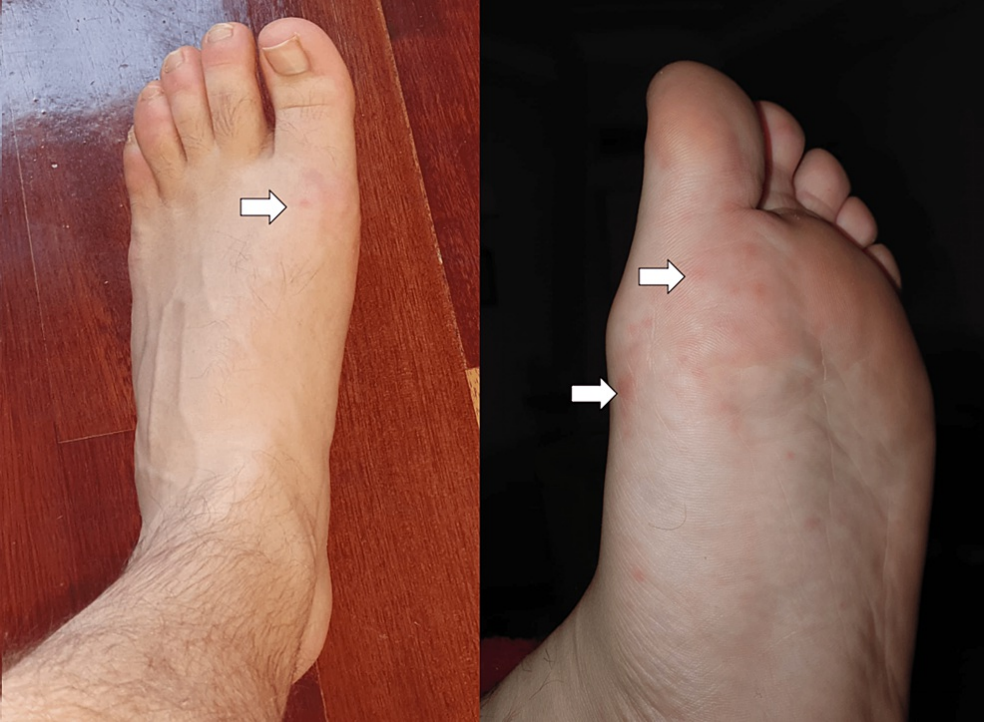
Macular lesions on the feet

**Figure 3:**
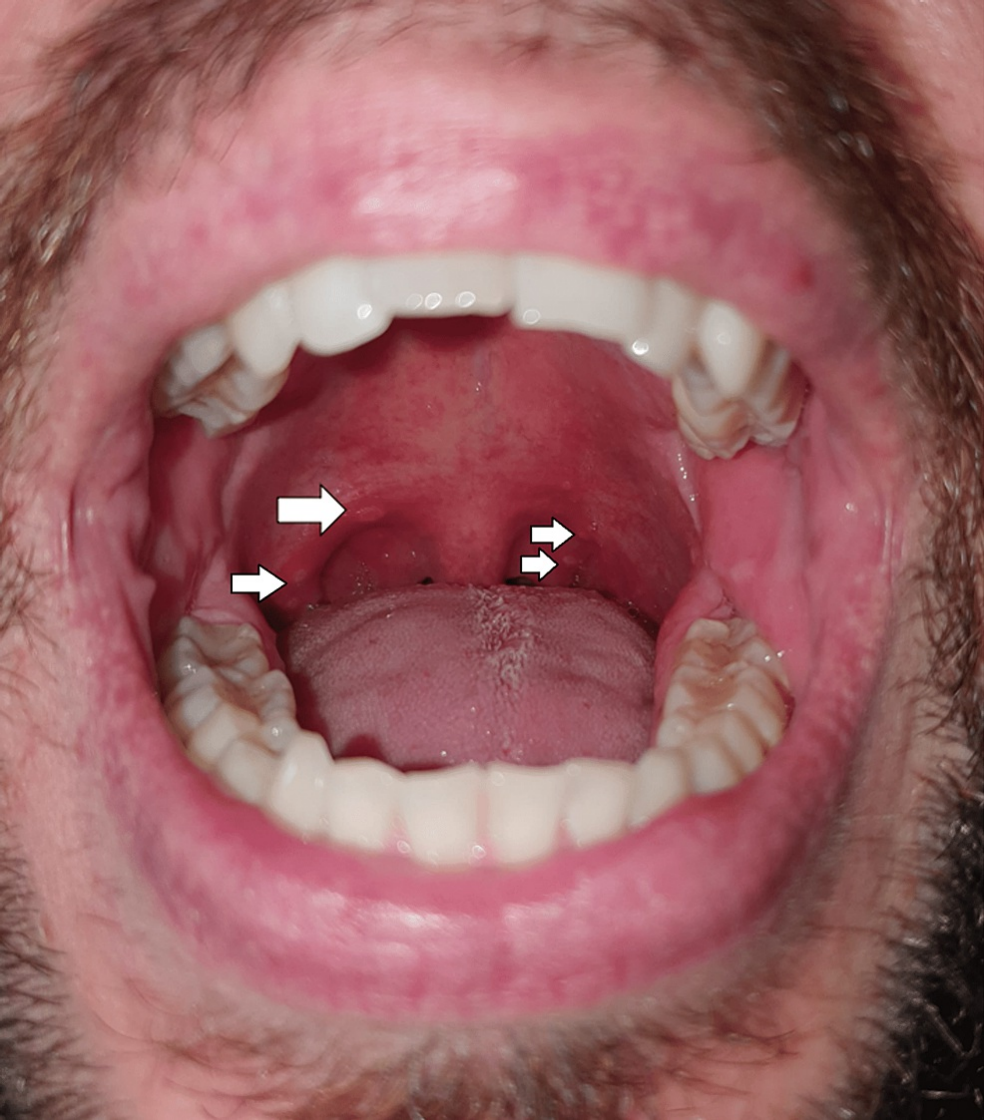
Hyperaemia and ulcerative lesions (white arrows)

**Figure 4:**
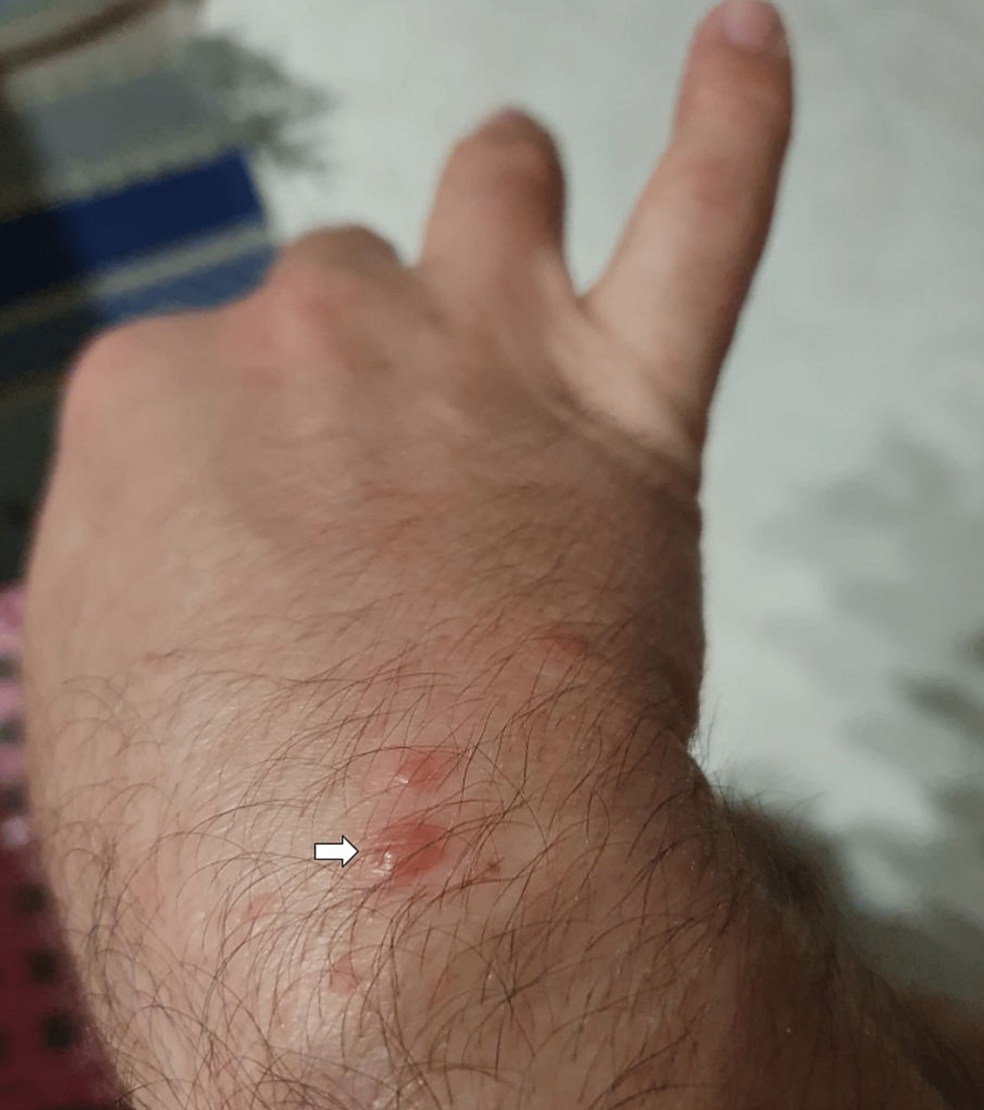
Bulla (dorsum of the hand)

**Figure 5:**
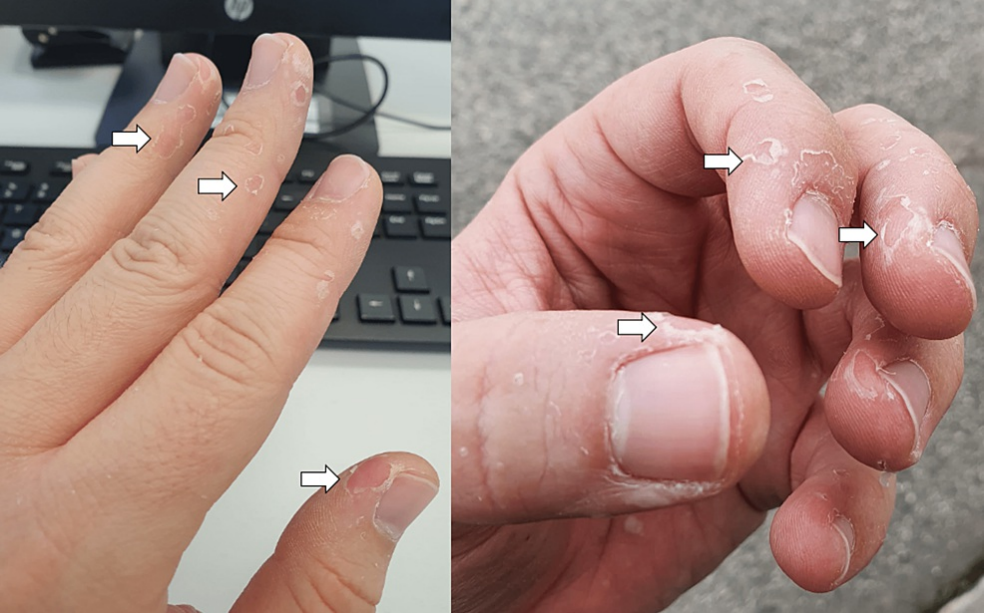
Skin desquamation

**Figure 6:**
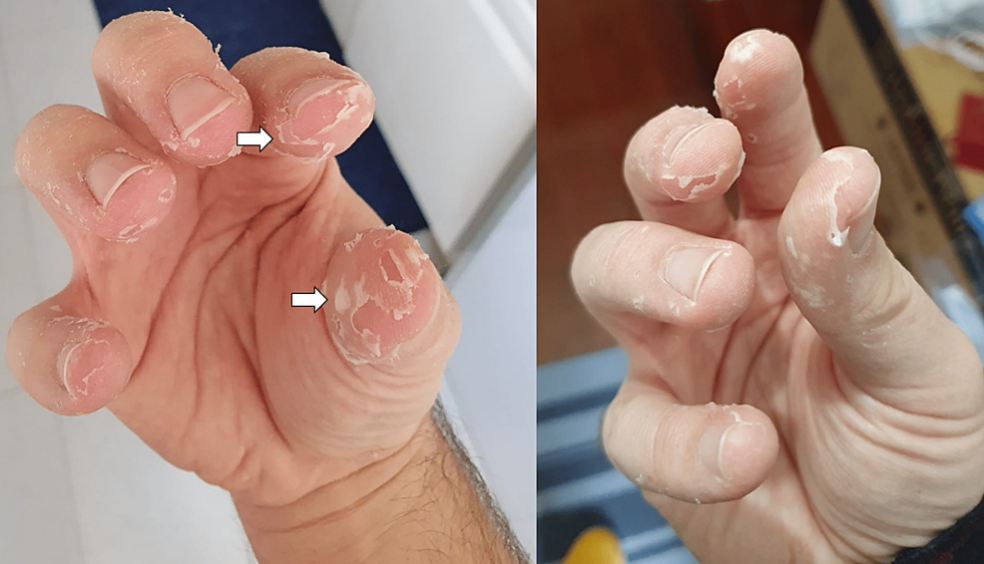
Skin desquamation in distal extremities of all fingers

The patient initially developed general malaise, prostration, myalgias, and subfebrile temperature within a week of evolution. The symptomatology progressed to headache and fever (axillary temperature 39°C). Concurrently, there was an emergence of macular lesions on the palm and sole of the feet and ulcers in the oral cavity. The macular lesions (Figures 1, 2) were associated with burning pain and paresthesias, leading to functional impotence. The ulcers were present in the oral cavity (Figure 3) and oropharynx, conditioned odynophagia, and dysphagia. The patient's children were diagnosed with HFM disease about a week before the onset of symptoms. The patient self-medicated with sucralfate, paracetamol, and hydration, with partial relief of symptoms. The symptoms persisted for about 10 days, with subsequent progressive improvement. It is worth highlighting the evolution of the skin lesions to bullae and skin desquamation (Figures 4-6) followed by resolution took about 15 days.

## Discussion

The typical presentation in children is an initial flu-like illness, which is later associated with painful ulcers in the oral cavity and painful vesicles on the palms and soles of the feet. In the adult population, more cases have been described in the last 10 years [2,5]. The diagnosis of HFMD in adults is imminently clinical since virus culture and laboratory identification requires specialized laboratories [5,6].

In the case presented, the transmission was through contact with the children, whose infection had been previously documented. In the study by Flipo et al., all patients were infected by very close relations [1]. The main differential diagnoses to consider in maculopapular rash are anaphylaxis, drug-induced, viral rashes, bacterial rashes, rickettsiosis, rheumatological pathology, and systemic pathology. Concerning infectious causes, the authors highlight chickenpox, monkeypox, rickettsiosis, and syphilis. As already mentioned, the patient had a history of chickenpox in childhood and did not present an itchy rash, lesions on the trunk, lesions at different stages, or pustules. Regarding monkeypox, the lesions did not evolve into pseudo-pustules and did not present umbilication, and the patient did not present anogenital or perioral lesions. The patient did not have contact with ticks, fleas, mites, or domestic animals, and his lesions didn't present the characteristic tache noire lesion, making rickettsiosis less likely. Lastly, with regard to secondary syphilis, most patients have lymph node enlargement with palpable nodes present in the posterior cervical, axillary, inguinal, and femoral regions, with a diffuse rash, and involvement of the trunk besides involvement of the extremities.

In recent years, the description of cases in adults has increased [3,7], going from typical presentation (similar to children) to atypical presentation (which makes diagnosis difficult). The usual presentation is characterized by a typical vesicular eruption on the hands, feet, and oral mucosa, and fever [7]. The atypical one may present with pseudo-purpuric lesions invading the epidermis, affecting other areas such as the scalp [1], face, limbs, and trunk, and high fever [7]. As reported by Cunha et al., infections of the central nervous system and myopericarditis may also arise [4]. As in children, maculopapular and vesicular erythematous lesions initially appear that can desquamate and evolve into superficial ulcerations [7].

## Conclusions

Fever and rash are common clinical presentations making the differential diagnosis list long, including infectious and non-infectious causes. Taking into account the multiplicity of etiologies, the clinical history, and epidemiological context become essential for a correct diagnosis. In this case, the epidemiological context was decisive. The HFMD presentation in adults should be part of the differential diagnosis of adults with rash, taking into account the increase in reports of these cases. The presentation in adults can be heterogeneous, making the diagnosis more difficult, hence the importance of recognizing this etiology.
